# Leucine 232 and hydrophobic residues at the ribosomal P stalk binding site are critical for biological activity of ricin

**DOI:** 10.1042/BSR20192022

**Published:** 2019-10-15

**Authors:** Yijun Zhou, Xiao-Ping Li, Jennifer N. Kahn, John E. McLaughlin, Nilgun E. Tumer

**Affiliations:** Department of Plant Biology, School of Environmental and Biological Sciences, Rutgers University, New Brunswick, NJ 08901-8520, U.S.A.

**Keywords:** protein-protein interactions, ribosomal P stalk, ribosome inactivating protein, ricin, sarcin ricin loop, toxin

## Abstract

Ricin interacts with the ribosomal P stalk to cleave a conserved adenine from the α-sarcin/ricin loop (SRL) of the rRNA. Ricin toxin A chain (RTA) uses Arg^235^ as the most critical arginine for binding to the P stalk through electrostatic interactions to facilitate depurination. Structural analysis showed that a P2 peptide binds to a hydrophobic pocket on RTA and the last two residues form hydrogen bonds with Arg^235^. The importance of hydrophobic residues relative to Arg^235^ in the interaction with the P stalk *in vivo* and on the toxicity of RTA is not known. Here, we mutated residues in the hydrophobic pocket to analyze their contribution to toxicity and depurination activity in yeast and in mammalian cells. We found that Leu^232^, Tyr^183^ and Phe^240^ contribute cumulatively to toxicity, with Leu^232^ being the most significant. A quadruple mutant, Y183A/L232A/R235A/F240A, which combined mutations in critical hydrophobic residues with R235A completely abolished the activity of RTA, indicating that Arg^235^ and hydrophobic residues are required for full biological activity. Y183A and F240A mutants had reduced activity on RNA, but higher activity on ribosomes compared with R235A *in vitro*, suggesting that they could partially regain activity upon interaction with ribosomes. These results expand the region of interaction between RTA and the P stalk critical for cellular activity to include the hydrophobic pocket and provide the first evidence that interaction of P stalk with the hydrophobic pocket promotes a conformational rearrangement of RTA to correctly position the active site residues for catalytic attack on the SRL.

## Introduction

Ricin, produced by the castor bean plant, is a type II ribosome inactivating protein (RIP), which consists of a ricin toxin A chain (RTA) and a ricin toxin B chain (RTB) connected by a disulfide bond. RTB is a lectin that can bind to glycoprotein or glycolipids on the surface of the cell membrane and facilitate endocytosis of the toxin [1]. Once inside the cell, ricin traffics to the endoplasmic reticulum (ER). The disulfide bond is reduced in the ER, releasing RTA from RTB [2]. The free RTA gets out of the ER through the ER-associated degradation (ERAD) pathway and becomes fully active in the cytosol [3]. Other type II RIPs such as abrin and Shiga toxin also have an enzymatically active A chain linked to a B chain [4]. In contrast, type I RIPs, such as pokeweed antiviral protein (PAP), saporin, gelonin and trichosanthin (TCS), contain only one enzymatically active chain [4]. RIPs depurinate a conserved adenine (A4324 in rat) in the α-sarcin/ricin loop (SRL) of the large rRNA [5], inhibiting interaction of the SRL with the elongation factors [6–8].

We previously showed that RTA binds to the ribosomal P stalk to depurinate the SRL [9–12]. The conserved C-termini of P proteins are critical for RTA–stalk interaction [13–15]. Peptides corresponding to C-terminal residues of P2 also interact with several other RIPs, including TCS [16], maize RIP (MOD) [17] and Shiga toxin [18–20]. The ribosomal stalk is a protein complex, which is part of the GTPase Associated Center (GAC) near the SRL [21]. The elongation factors bind to the C terminal domain (CTD) of the stalk proteins [22,23] and their GTPase activities are dependent on the presence of the stalk [22,23]. The bacterial and eukaryotic stalk protein CTDs are structurally distinct [24] and are responsible for differentiating between the prokaryotic and eukaryotic elongation factors [22,24–26]. In eukaryotes, the stalk is a pentameric protein complex formed by a P0 protein and two P1-P2 heterodimers [27,28]. The CTD of P0, P1 and P2 proteins, especially their last 11 residues [SDDDMGFGLFD], are highly conserved among the eukaryotes [29].

The stalk binding site of RTA is covered by RTB in the holotoxin. This area is located on the opposite side of the active site (Tyr^80^, Tyr^123^, Glu^177^, Arg^180^ and Trp^211^) [30]. Since the ribosome binding site is blocked by RTB ricin holotoxin can only depurinate the free rRNA, but cannot bind or depurinate the ribosome [31]. Ricin binds the ribosome by a two-step mechanism, which involves first slow and non-stalk specific electrostatic interactions with the ribosome, followed by fast electrostatic interactions with the P stalk [10]. We mutated arginine residues at the RTA/RTB interface and showed that Arg^235^ is the most important arginine residue at the P stalk binding site and acts as an anchor residue to bind to P proteins [15]. The R235A mutation and the double, triple and quadruple mutations, which combined R235A with R189A and R193A, reduced the toxicity and the depurination activity of RTA [13,15]. The R189A, R193A, R234A single mutants lost the fast electrostatic interactions with the ribosome [10,15]. The interaction of R235A or the double mutants, R189A/R234A and R193A/R235A, with the ribosome was not detectable by Biacore analysis [13,15]. These data demonstrated that arginines are critical for the electrostatic interactions with the ribosome. The fast electrostatic interactions occur with the P stalk and are critical for the depurination activity and toxicity of RTA [13,15]. Arg^235^ cooperates with nearby arginines, Arg^234^, Arg^189^, Arg^191^, Arg^193^, Arg^196^ and Arg^197^ to bind to the stalk via fast electrostatic interactions [32]. Although mutations in arginines reduced the depurination activity and toxicity of RTA, none of the single arginine mutations eliminated the toxicity of RTA completely even when they were combined [15].

**Figure 1:**
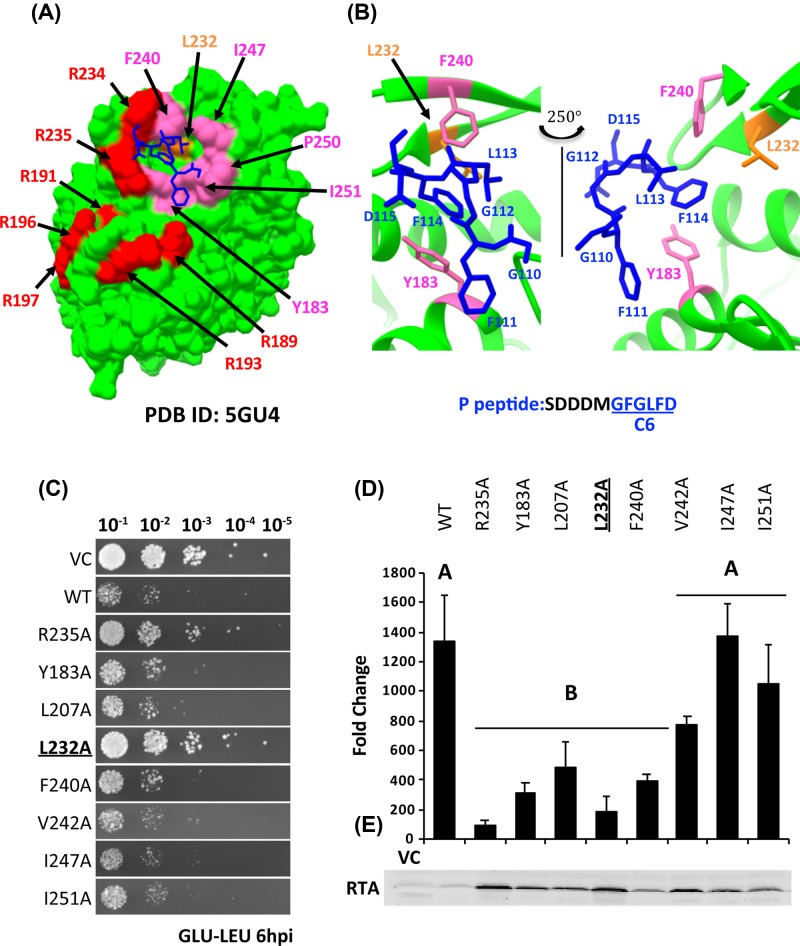
L232A is the least toxic hydrophobic mutant (**A**) Structure of RTA-P6 (PDB ID: 5GU4) visualized using UCSF Chimera [37]. RTA is colored green, Arg^189^, Arg^191^, Arg^193^, Arg^196^, Arg^197^, Arg^234^ and Arg^235^ are colored red; Tyr^183^, Phe^240^, Ile^247^, Ile^250^ and Pro^250^ are colored hot pink. Leu^232^ is colored orange. P6 peptide is colored blue. (**B**) Zoomed-in view of the hydrophobic pocket. (**C**) Viability assay of single mutants in yeast. After induction of toxin expression for 6 h, cells were spotted on SD-Leu medium containing glucose (GLU-LEU). (**D**) Depurination of single mutants in yeast at 0 hpi. Fold change in depurination is shown relative to the VC. Data were analyzed by Fisher’s LSD test for statistical differences. Means denoted by different letters indicate significant differences between treatments (*P*<0.001). (**E**) RTA expression in yeast at 8 hpi. Total protein stain (Supplementary Figure S1C) was used as the loading control. Abbreviations: LSD, least significant difference; VC, vector control/empty vector.

When RTA was co-crystallized with a peptide corresponding to the last 11 (P11) residues of P2 or a 10-mer P2 peptide (P10) that was fused to the C-terminus of RTA, only the last six residues [GFGLFD] (P6) bound to a hydrophobic pocket formed by Tyr^183^, Leu^207^, Leu^232^, Phe^240^, Val^242^, Ile^247^, P^250^ and Ile^251^ (Figure 1A,B) was observed in the structure [33,34]. The structure analysis validated the importance of Arg^235^ and showed that it forms hydrogen bonds with the last two residues (Phe^114^ and Asp^115^) of the P2 peptide [33,34]. Interaction with the acidic residues at the N-terminus of P11 [SDDDM] was not defined in either structure even though biochemical analysis indicated that this motif is important for ribosome recognition of RTA to facilitate depurination [15,34,35]. Alanine substitutions at Tyr^183^ and Phe^240^ affected the interaction of P10 with RTA *in vitro* [33]. Hydrophobic residues that are critical for ribosome binding, depurination activity and toxicity of RTA *in vivo* have not been identified and relative importance of the hydrophobic residues compared with Arg^235^ is not known. Here we identify the hydrophobic residues critical for binding to the stalk in mammalian cells and in yeast and show that the toxicity and the activity of RTA can be eliminated by combining mutations in Arg^235^ with mutations in critical hydrophobic residues without altering the active site.

## Materials and methods

### Protein structure visualization

All protein structures were downloaded from Protein Data Bank (PDB) [36] and visualized using the UCSF Chimera package [37]. Protein structure alignment was performed using the Needleman–Wunsch algorithm in MatchMaker function based on the secondary structure.

### Cloning

Site-directed mutagenesis was carried out on mature RTA (mRTA) without the 35 residue N-terminal leader previously cloned into the pBLUEscript plasmid (NT855) to generate RTA variants Y183A (NT1737), L207A (NT1734), F240A (NT1736), V242A (NT1824), I247A (NT1825), I251A (NT1826), L232A/R235A (NT1756), F240A/Y183A (NT1757), Y183A/R235A/F240A (NT1823), Y183A/L232A/F240A (NT1827) and Y183A/L232A/R235A/F240A (NT1833). For yeast experiments, toxin genes were cloned into the yeast expression vector pRS415 with *LEU2* selective marker under the control of *GAL1* promoter between XbaI and XhoI: Y183A (NT1749), L207A (NT1746), F240A (NT1748), V242A (NT1829), I247A (NT1832), I251A (NT1830), L232A/R235A (NT1758), F240A/Y183A (NT1759), Y183A/R235A/F240A (NT1828), Y183A/L232A/F240A (NT1831) and Y183A/L232A/R235A/F240A (NT1834). For protein purification, toxin genes were subcloned from the yeast expression vector into the NcoI and XhoI sites of the pET28 protein expression vector with a 10× His-tag at their N-terminus to generate wild-type (WT) RTA (NT1430), Y183A (NT1773) and F240A (NT1772). For mammalian experiments, toxin genes were cloned into the pCAGGS (NT1581) mammalian expression vector [38]. WT mRTA expressing plasmid (AJ4, NT1849) [39] was a gift from Dr. Wendie S. Cohick. RTA variants R235A (NT1852), L232A (NT1853), L232A/R235A (NT1851), Y183A/L232A/F240A (NT1854) and Y183A/L232A/R235A/F240A (NT1855) were made by site-directed mutagenesis (Genewiz, Piscataway, NJ). All mutations were confirmed by sequencing.

### Yeast transformation and culture

*Saccharomyces cerevisiae* strain W303 (*MATa ade2-1 trp1-1 ura3-1 leu2-3, 112 his-3-11, 15 can1-100*) was transformed with the empty vector (NT1616), WT RTA (NT1622) or RTA variants and plated on SD-Leu supplemented with 2% glucose (Glu, -Leu) at 30°C for 48 h. Colonies were inoculated in Glu, -Leu and grew overnight at 30°C for 17 h (0 hpi) before induction. For induction, three OD of cells were collected and switched to 10 ml Gal, -Leu medium at 0.3 OD/ml and incubated at 30°C for indicated hours.

### Yeast viability assay

Yeast cells were harvested after induction for the indicated hours and adjusted to 10^−1^ OD/ml in Glu, -Leu medium and serial dilutions (10^−1^, 10^−2^, 10^−3^, 10^−4^, 10^−5^) were made. A total of 10 μl of each dilution was spotted on the indicated medium incubated in 30°C for 24–48 h.

### Western blot analysis

For yeast Western blot analysis, total protein was extracted as described [40]. Cells (OD_600_ of 3) were washed with 2 M LiAc on ice for 5 min, then by 0.4 M NaOH on ice for 5 min followed by 0.5 ml 100 mM Tris/HCl pH 6.8 and spun down at 6000×*g*. The pellet was resuspended in 1× SDS/PAGE sample loading buffer (15 μl/OD) and boiled for 5 min at 95°C followed by incubation in 30°C for 1 h. Samples were pelleted and 15 μl of the supernatant was loaded on SDS/PAGE. For mammalian Western blot, 1 × 10^5^ cells from one well of a 12-well plate was scraped off the plate at 22 h after transfection and washed with 500 μl of cold 1× PBS. Cells were spun down at 2000 rpm then lysed with 100 μl lysis buffer (1% Triton X-100, 80 mM β-glycerophosphate, 50 mM HEPES, 2 mM EDTA, 2 mM EGTA, 10 mM sodium fluoride, 0.1% SDS, 1 mM PMSF, 2 mM sodium orthovanadate) [41] supplemented with 1× Roche cOmplete Protease Inhibitor Cocktail (Roche Diagnostics GmbH, Mannheim, Germany) and 1 μl Benzonase Nuclease (EMD Millipore Corp., Billerica, MA, U.S.A.). The supernatant (30 μl) was loaded on a precast 4–20% SDS/PAGE gel (Genscript, Piscataway, NJ). Proteins were transferred to Trans-Blot Turbo Transfer Pack 0.2 μm nitrocellulose membrane (Bio-Rad Laboratories, Hercules, CA), dried and then stained for total protein using REVERT total protein stain kit (Li-COR, Lincoln, NE, U.S.A.). Before blocking, the total protein stain was stripped using the reversal buffer provided in the REVERT total protein stain kit (Li-COR, Lincoln, NE, U.S.A.). The blocked membrane was probed with 1:5000 diluted PB10 [42,43] antibody overnight at 4 °C followed by 1:10000 diluted Li-COR IRDye 800CW goat anti-Mouse antibody (Li-COR, Lincoln, NE, U.S.A.) for 1 h in room temperature with 3 × 10 min washes in between. The membrane was scanned using Odessey Clx imager (Li-COR, Lincoln, NE, U.S.A.). Novex Sharp Prestained Protein Standard (Cat# LC5800, Invitrogen, Carlsbad, CA, U.S.A.) was used as ladder.

### Depurination assay

The level of depurination was measured by qRT-PCR as previously described [44]. Briefly, for yeast and mammalian cell depurination, RNA was extracted using an RNase Mini Kit from QIAGEN (Valencia, CA, U.S.A.). For *in vitro* ribosome depurination, 4 pmol of purified yeast ribosomes were treated with purified toxin in reaction buffer (60 mM KCl, 10 mM Tris/HCl and 10 mM MgCl_2_, pH 7.4) and incubated at room temperature for 5 min. The rRNA was extracted by phenol/chloroform followed by ethanol precipitation. For total RNA depurination, 1 μg of yeast total RNA was treated with toxin at indicated concentrations in reaction buffer (20 mM citrate, pH 5) then RNA was precipitated with ethanol. Extracted RNA (375 ng) was used for reverse transcription using a High Capacity cDNA Reverse Transcription Kit from Applied Biosystems (Thermo Fisher, Waltham, MA) to generate cDNA. Reverse transcription product was diluted 1:200 fold with ddH_2_O then 4 μl was used in real-time PCR. For yeast *in vivo* or *in vitro* ribosome depurination, Dep-For2: 5′-ACTAATAGGGAACGTGAGCTG-3′ and Dep-Rev: 5′-CCGAATGAACTGTTCCACA-3′ were used as primers to detect depurinated rRNA. 25S-For: 5′-AGACCGTCGCTTGCTACAAT-3′ and 25S-Rev: 5′-ATGACGAGGCATTTGGCTAC-3′ were used as primers to detect total 25S rRNA. For mammalian cell *in vivo* depurination, hDep_F: 5′- TGCCATGGTAATCCTGCTCAGTA-3′ and hDep_R: 5′- TCTGAACCTGCGGTTCCACA-3′ were used as primers to detect depurinated rRNA. h28S_F: 5′-GATGTCGGCTCTTCCTATCATTGT-3′ and h28S_R: 5′-CCAGCTCACGTTCCCTATTAGTG-3′ were used as primers to detect total 28S rRNA. Quantification was done using the comparative *C*_T_ method to calculate fold change of depurination relative to vector control/empty vector (VC).

### Mammalian cell culture and transfection

Vero cells CCL-81 (ATCC, Manassas, VA) were maintained in Dulbecco’s Modified Eagle’s Medium (DMEM) supplemented with high glucose (4.5 g/l), glutamine, Phenol Red, sodium pyruvate (Gibco, Life Technologies Limited, Paisley, U.K.), 10% fetal bovine serum (ATCC, Manassas, VA) and 100 I.U./ml Penicillin and 100 μg/ml Streptomycin (ATCC, Manassas, VA) at 37°C in 5% CO_2_. At confluency, cells were trypsinized, diluted to 2 × 10^6^ cells/ml with complete medium and a total of 10 ml of cell culture was transferred to a 75-cm^2^ flask. For transfection, 100 μl cells were seeded at 1 × 10^5^ in a 96-well plate and incubated for 24 h to 80% confluence, then the medium was replaced by 50 μl medium lacking fetal bovine serum and preincubated for 10 min with 0.3 μl Lipofectamine 2000 (Invitrogen, Thermo Fisher, Carlsbad, CA) and 50 ng of each of RTA or EGFP expressing plasmid (pEGFP-N1). After 2 h of incubation at 37°C in 5% CO_2_, transfection mixture was replaced by medium with 10% serum and further incubated for 22 h before EGFP analysis.

### Mammalian translation inhibition assay

Vero cells CCL-81 (ATCC, Manassas, VA) were co-transfected with RTA plasmids and EGFP expressing plasmid, pEGFP-N1 (NT1580) and plated in a black clear-bottom 96-well plate. At 22 h after transfection, GFP fluorescence was quantified in a Biotek (BioTek Instruments, Inc., Winooski, VT) plate reader with 485/20 excitation filter and 516/20 emission filter. Assay was performed in quadruplicate. Fluorescence from cells co-transfected with pCAGGS (Vector control, VC, NT1581) and pEGFP-N1 (NT1580) was used as the positive control and cells without pEGFP-N1 as the negative control.

### mRNA quantification in mammalian cells

Vero cells CCL-81 (ATCC, Manassas, VA) were co-transfected with RTA plasmids and EGFP plasmid, pEGFP-N1 (NT1580) and plated in a black clear-bottom 96-well plate. At 22 h after transfection, total RNA was extracted using an RNase Mini Kit from QIAGEN (Valencia, CA, U.S.A.). Total RNA (375 ng) was used in reverse transcription using a High Capacity cDNA Reverse Transcription Kit from Applied Biosystems (Thermo Fisher, Waltham, MA) to generate cDNA. Reverse transcription product was diluted at 1:200 fold with ddH_2_O then 4 μl was used in real-time PCR. mRTA_2F: 5′-ATCCTGGTCGAGCTCAGTAACC-3′ and mRTA_2R: 5′-CACGTCCAGGGCCAGAGT-3′ were used as primers to detect RTA mRNA. h28S_F: 5′-GATGTCGGCTCTTCCTATCATTGT-3′ and h28S_R: 5′-CCAGCTCACGTTCCCTATTAGTG-3′ were used as primers to detect 28S rRNA. Quantification was done using the comparative *C*_T_ method to calculate the fold change in mRNA levels relative to the VC.

### Protein purification

WT and RTA variant protein expression plasmids were transformed into BL21-CodonPlus(DE3)-RIPL (F^–^ *ompT hsdS*(r_B_^–^ m_B_^–^) *dcm^+^* Tet^r^ gal λ(DE3) *endA Hte* [*argU proL* Cam^r^] [*argU ileY leuW* Strep/Spec^r^]) and plated on MDG (25 mM Na_2_HPO_4_, 25 mM KH_2_PO_4_, 5 mM Na_2_SO_4_, 2 mM MgSO_4_ and 50 mM NH_4_Cl, 0.25% aspartate, 0.2× trace metal and 0.5% glucose) plate with 2% agar [45]. Cells from a single colony were inoculated into 1 ml MDG medium for 8 h then into 200 ml MDG medium overnight before inoculation into TPG (2% tryptone, 0.2% Na_2_HPO_4_, 0.1% KH_2_PO_4_, 0.8% NaCl, 1.5% yeast extract and 0.2% glucose) medium. Pellet from every 1L TPG culture was resuspended in 50 ml lysis buffer consisting of buffer A (20 mM Tris/HCl, pH8.5, 1 mM DTT and 1L Glycerol) supplemented with 0.01% Benzonase Nuclease (EMD Millipore Corp., Billerica, MA, U.S.A.), 1 tablet/50 ml Roche cOmplete™ Protease Inhibitor (Roche Diagnostics GmbH, Mannheim, Germany) and 200 μg/ml fresh lysozyme (MP Biomedicals, LLC., Solon, OH) and lysed by sonication before column purification. WT RTA (NT1430) (2 L) was loaded on a 2× HiTrap Q HP 5 ml column (GE Healthcare Bio-Sciences, Uppsala, Sweden) and eluted with buffer B (20 mM Tris/HCl, pH 8.5, 1 mM DTT and 5% Glycerol, 2 M NaCl). Protein containing fractions were pooled, adjusted to the same buffer as buffer 20 (20 mM Tris/HCl, pH 8.5, 0.5 M NaCl, 20 mM Imidazole, 1 mM DTT and 5% Glycerol), loaded on to HisTrap Crude FF 5 ml (GE Healthcare Bio-Sciences, Uppsala, Sweden) followed by elution with buffer 500 (20 mM Tris/HCl, pH 8.5, 0.5 M NaCl, 500 mM Imidazole, 1 mM DTT and 5% Glycerol). The eluted protein was pooled and dialyzed against the dialysis buffer (20 mM Tris/HCl, pH 8.5, 0.1 M NaCl, 2 mM TCEP and 5% Glycerol), concentrated using Amicon Ultracel-3K Centrifugal Filters (Merck Millipore Ltd., Tullagreen, Carrigtwohill, Co. Cork, IRL) and frozen in −80°C. Y183A (NT1773) was purified similar to WT RTA, except 6 L of cell pellets were lysed. F240A (NT1772) was also purified similarly, except 8 L of cell pellets were lysed and an 80 ml Q Sepharose FF column was used as the first column and fractions after the HisTrap column were loaded on to Heparin HP 1 ml (GE Healthcare Bio-Sciences, Uppsala, Sweden) with buffer C (20 mM Tris/HCl, pH 8.5, 0.1 mM EDTA, 1 mM DTT and 5% Glycerol) as binding buffer and buffer D (20 mM Tris/HCl, pH 8.5, 0.1 mM EDTA, 1 mM DTT, 1 M NaCl and 5% Glycerol) as elution buffer.

### Ribosome interaction

Ribosome interaction was analyzed on Biacore T200 (GE Healthcare, Pittsburg, PA, U.S.A.). WT RTA and RTA variants were immobilized on an NTA chip at 1000 response unit (RU). R193A/R235A (NT1414), which does not bind to the ribosome [13] was captured on reference channel; 5, 10, 20 nM ribosomes were passed over the surface of the chip as the analyte at 40 μl/min for 2 min followed by 5-min dissociation. The chip was regenerated by 1-min injection of 350 mM EDTA at pH 8 and 1-min injection of 0.3% SDS. The running buffer contained 10 mM HEPES, pH 7.4, 150 mM NaCl, 50 μM EDTA, 0.003% P20 and 5 mM MgCl_2_.

### Protease digestion

GluC and TPCK treated trypsin (Trypsin-ultra, MS grade) were purchased from New England Biolabs (Ipswich, MA, U.S.A.). Four microgram of protein was incubated with 0.2 μg of Glu-C (1:20) in Glu-C reaction buffer (50 mM Tris/HCl pH 8, 0.5 mM Glu–Glu dipeptide) for 4 h at 37°C. Ten microgram of protein was incubated with 0.25 μg of Trypsin (1:40) in trypsin reaction buffer (50 mM Tris/HCl pH 8, 20 mM CaCl_2_) for 5–30 min at 25°C. TLCK treated chymotrypsin was purchased from Thermo Fisher (Waltham, MA, U.S.A.). Ten microgram of protein was incubated with 0.25 μg of chymotrypsin (1:40) in buffer (100 mM Tris/HCl, pH 8, 10 mM CaCl_2_) for 1 h at 37 °C.

### Statistical analysis

Statistical analyses of data were conducted using SAS 9.4 [46]. One-way analyses of variance (ANOVA) using PROC ANOVA was used to test for significant treatment effects in the model (depurination as the dependent variable; block and treatment as the independent variables). Once a significant (*P*<0.05) treatment effect for the model was determined by ANOVA, the Fisher’s Least Significant Difference (LSD) was applied to test for statistical differences between treatments.

## Results

### L232A is the least toxic single hydrophobic mutant

To determine the relative contribution of the hydrophobic residues to the depurination activity and toxicity of RTA, Tyr^183^, Leu^207^, Leu^232^, Phe^240^, Val^242^, Ile^247^, P^250^ and Ile^251^, which interact with the last six residues of P proteins [33,34] (Figure 1A,B) were each replaced with alanine on the mRTA and cloned into the low copy yeast expression vector pRS415 under the control of the *GAL1* promoter. Toxicity of the mutants was examined in yeast by inducing toxin expression with galactose for 6 h (6 hpi) then by spotting on glucose plates (Figure 1C). The VC, WT RTA (WT) and R235A were used as controls. The L232A was the least toxic among the single hydrophobic mutants. Its toxicity was similar to R235A (Figure 1C). The depurination activity of the mutants was examined using qRT-PCR [44]. Due to the leakiness of the *GAL1* promoter, we were able to detect depurination of WT RTA in glucose medium (0 hpi) (Figure 1D). The depurination level of R235A, L232A, Y183A, L207A and F240A were significantly reduced compared with WT (*P*<0.001), while V242A, I247A or I251A did not show reduction (Figure 1D). L232A showed the lowest level of depurination among the hydrophobic mutants (Figure 1D). Western blot analysis showed that all mutants were expressed (Figure 1E and Supplementary Figure S1A). The total protein level was quantified by total protein stain (Supplementary Figure S1C). As previously observed [15] RTA expression levels (Figure 1E and Supplementary Figure S1A) inversely correlated with the toxicity and depurination level of RTA since a lower level of depurination allowed the mutant protein to accumulate with time. These data showed that the activity of Y183A, L207A, L232A and F240A was reduced compared with WT RTA and L232A was the least toxic and the least active hydrophobic mutant.

### Combining L232A, R235A, Y183A and F240A point mutations further reduces the toxicity of RTA

**Figure 2:**
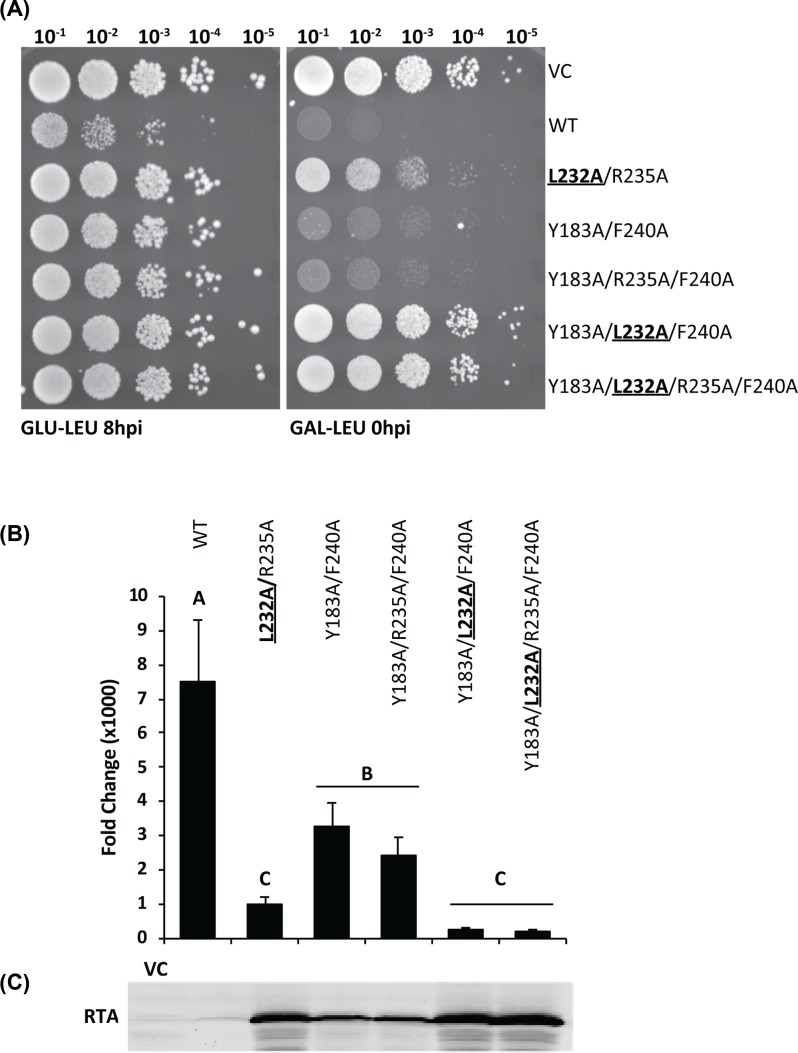
Double, triple and quadruple hydrophobic mutants show further reduction in toxicity (**A**) Viability assay of RTA mutants in yeast. An overnight yeast culture grown in SD-Leu medium containing galactose for 8 h was spotted on an SD-Leu plate containing glucose (GLU-LEU) or directly spotted on an SD-Leu plate containing galactose (GAL-LEU). (**B**) Depurination of RTA variants in yeast at 6 hpi measured by qRT-PCR. Fold change in depurination is shown relative to the VC. Data were analyzed by Fisher’s LSD test for statistical differences. Means denoted by a different letter indicate significant differences between treatments (*P*<0.05). (**C**) RTA expression in yeast at 8 hpi. Total protein stain (Supplementary Figure S1D) was used as the loading control.

Alanine substitutions at Tyr^183^ and Phe^240^ reduced the depurination level of RTA. Since Tyr^183^ and Phe^240^ likely form π–π stacking interactions with Phe^114^ at the P stalk CTD [SDDDMGFGL**F**D] (Figure 1B), we hypothesized that Tyr^183^ and Phe^240^ may compensate for each other when one or the other residue is mutated. Therefore, we combined the Y183A and F240A mutations to determine if the toxicity is further reduced. The triple mutant Y183A/L232A/F240A was also made to determine if combining these mutations with the L232A mutation will reduce the toxicity further. The double mutant, L232A/R235A, the triple mutant, Y183A/R235A/F240A and the quadruple mutant, Y183A/L232A/R235A/F240A were made to determine if mutations in critical hydrophobic residues can abolish toxicity when they are combined with the R235A mutation. The spot assay showed that the toxicity of the different mutant combinations was reduced compared with WT and their viability remained similar to the VC even after 8 h of induction on galactose followed by spot dilution on glucose plates (Figure 2A, GLU-LEU). These results showed that the multiple mutants were less toxic than the single mutants (Figure 1C). When viability of these mutants was examined by directly spotting them on galactose plates the double and triple mutants that contained the L232A mutation grew, while the double and triple mutations that did not contain L232A did not grow (Figure 2A, GAL-LEU). The triple, Y183A/L232A/F240A and the quadruple, Y183A/L232A/R235A/F240A mutants grew even better than the double mutant, L232A/R235A, indicating that while Leu^232^ is the most critical residue, and all three hydrophobic residues contribute cumulatively to toxicity.

Depurination by the multiple mutants was not detectable in yeast at 0 hpi (Supplementary Figure S2A,B), indicating that their activity was reduced compared with the single mutants. The level of depurination at 6 hpi (Figure 2B) correlated inversely with the Western blot results (Figure 2C and Supplementary Figure S1B), which were quantified by total protein stain (Supplementary Figure S1D). The double, triple and quadruple mutants were significantly less active than WT RTA and expressed more protein. The double, triple and quadruple mutants that contained L232A were less toxic and depurinated less than Y183A/F240A and Y183A/R235A/F240A, which did not contain L232A (*P*<0.05), indicating that L232A mutation caused a further reduction in the depurination activity and toxicity of RTA when it was combined with mutations in the other hydrophobic residues.

### Y183A and F240A show higher activity on ribosomes than R235A

**Figure 3:**
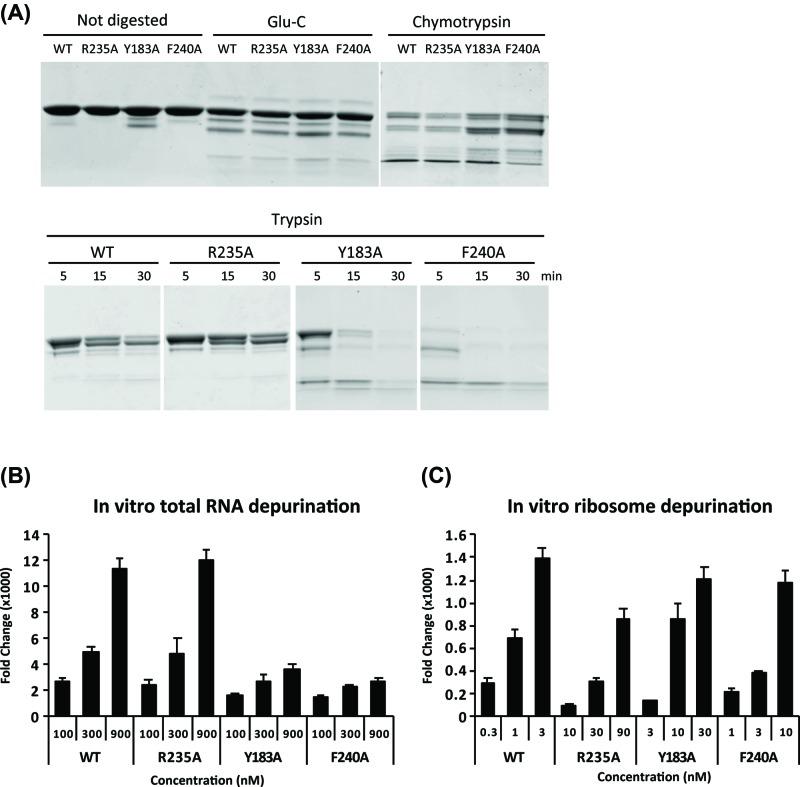
Y183A and F240A show higher activity on ribosomes than R235A *in vitro* (**A**) Protease digestion of purified WT RTA and mutant proteins. Four micrograms of each non-digested protein was loaded as control. For Glu-C, 4 μg of protein was digested for 4 h at 37°C. For chymotrypsin, 10 μg of protein was digested for 1 h at 37°C. For trypsin, 10 μg of protein was digested for 5–30 min as indicated at 25°C. (**B**) Depurination of yeast total RNA analyzed by qRT-PCR. WT RTA and mutants were used at 100, 300 and 900 nM as indicated. Results from a study of three independent experiments were analyzed by StepOnePlus Software from Applied Biosystems (Foster City, CA, U.S.A.). (**C**) Depurination of purified yeast ribosomes. Yeast ribosomes were treated with different concentrations of toxins as indicated. Results from a study of four independent experiments were analyzed by StepOnePlus Software from Applied Biosystems (Foster City, CA, U.S.A.).

In order to further characterize these mutants, we expressed the WT, Y183A and F240A in *Escherichia coli* (*E. coli*) and purified them to 90% homogeneity (Supplementary Figure S3A). We also attempted purification of L232A, L232A/R235A and F240A/Y183A. However, we could not see any protein expressed in *E. coli* (Supplementary Figure S3B) and could not obtain pure protein. To determine if Y183A and F240A folded properly, we digested the recombinant proteins with Glu-C, chymotrypsin and trypsin (Figure 3A). Glu-C selectively cleaves peptide bonds at the C-termini of glutamic acid residues and at 100–300 times slower rate at the C-termini of aspartic acid residues. Chymotrypsin selectively cleaves at the C-termini of tyrosine, phenylalanine, tryptophan and leucine residues. Trypsin selectively cleaves at the C-termini of lysine and arginine residues. R235A showed similar digestion pattern as WT with all three proteinases. The Glu-C and chymotrypsin digestion patterns of Y183A and F240A were the same as WT and R235A. However, they were digested much faster by trypsin than WT or R235A (Figure 3A). These results indicated that folding of Y183A and F240A was affected, resulting in a difference in the exposure of lysine and arginine residues to trypsin compared with WT and R235A.

However, the significant biological activity of Y183A and F240A in whole cells suggested that the mutations did not abolish their function *in vivo*. We examined the activity of recombinant Y183A and F240A on total RNA from yeast (Figure 3B) and on purified yeast ribosomes (Figure 3C). Depurination activity on naked RNA depends on the enzymatic activity carried out by the active site residues (Tyr^80^, Tyr^123^, Glu^177^, Arg^180^ and Trp^211^). Since there is no interaction with the P stalk to help RTA bind to the SRL, RNA depurination occurs at pH 5. In contrast, when purified ribosomes are used as the substrate, the depurination activity is stimulated by P stalk binding and the depurination occurs at the physiological pH. Thus, by comparing activities of the mutants on naked RNA versus on purified ribosomes, we can determine how interaction with the ribosomal proteins affects the depurination activity of RTA mutants. R235A showed the same activity on naked RNA as WT, while its activity on salt-washed yeast ribosomes was reduced by 80-fold [15]. The activity of Y183A and F240A on naked RNA did not show significant difference compared with WT and R235A at 100 nM RTA concentration, but at 900 nM, it was reduced more than three-fold compared with WT and R235A (Figure 3B). The activity of Y183A and F240A on purified yeast ribosomes was reduced by only nine- and three-fold, respectively compared with WT while the activity of R235A was reduced 80-fold compared with WT (Figure 3C). These results demonstrated that although Y183A and F240A had lower activity on naked RNA than R235A, they had higher activity on ribosomes compared with R235A.

**Figure 4:**
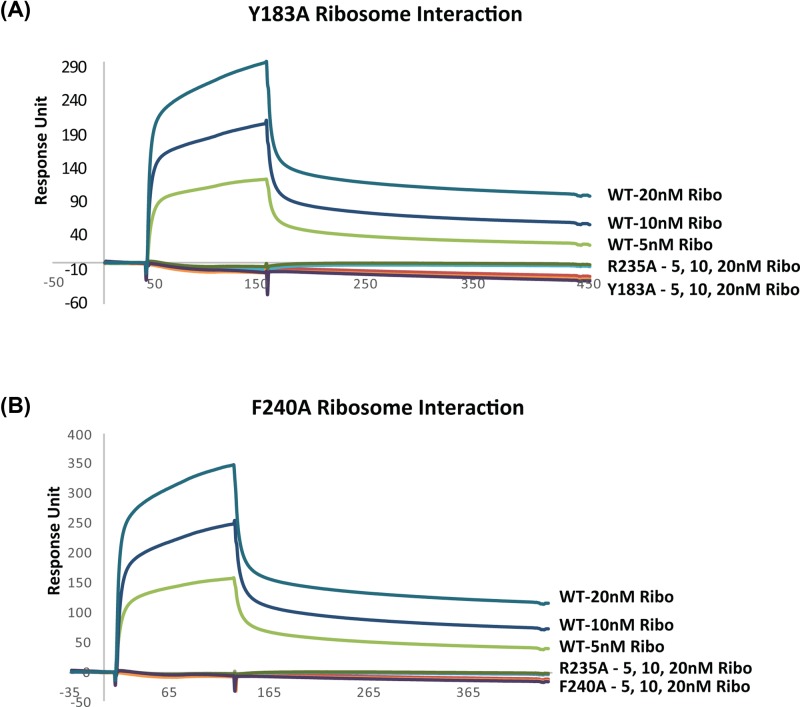
Interaction of Y183A and F240A with the ribosome by SPR Toxins were captured on an NTA chip of a Biacore T200. R193A/R235A was captured on Fc1 at 1080 RU as a reference. R235A was captured on Fc2 at 995 RU. (**A**) Y183A was captured on Fc3 at 957 RU. (**B**) F240A was captured on Fc3 at 900 RU. WT RTA was captured on Fc4 at 951 RU. Ribosomes were passed over surface of the chip at 5, 10 and 20 nM as the analyte. The running buffer contained 10 mM HEPES, pH 7.4, 150 mM NaCl, 5 mM MgCl_2_, 50 μM EDTA and 0.003% Surfactant P20. The interaction was measured at 25°C. The signals were normalized to the ligand level of 1000 RU. The experiment was repeated three times.

To determine if Y183A and F240A can bind ribosomes better than R235A, we examined ribosome binding using surface plasmon resonance (SPR) with a Biacore T200. Although WT RTA showed concentration-dependent binding to the ribosome, no interaction with the ribosome was detected with either Y183A (Figure 4A) or F240A (Figure 4B). As previously reported [15], R235A did not show detectable binding to the ribosome. Since Y183A and F240A could depurinate the ribosome *in vitro* (Figure 3C), they likely interacted with the ribosome at a level, which was outside the limit of detection of the method we used.

### Combining L232A, R235A, Y183A and F240A eliminates the activity of RTA in Vero cells

**Figure 5:**
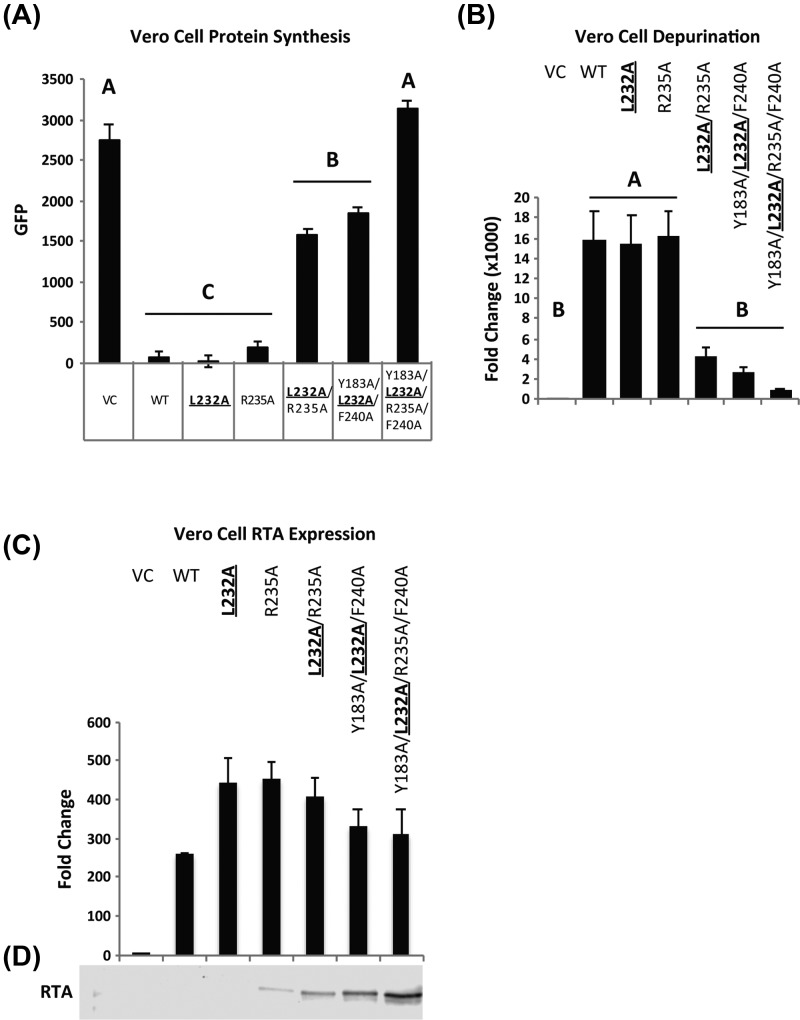
Hydrophobic mutants show reduced activity and toxicity in Vero cells (**A**) Protein synthesis in Vero cells co-transfected with plasmids containing VC, WT RTA or RTA mutants and pEGFP-N1. Fluorescence was measured at 22 h after transfection. The experiment was repeated four times. Data were analyzed by Fisher’s LSD test for statistical differences. Treatments in different groups (A–C) are statistically different from each other (*P*<0.001). (**B**) Ribosome depurination in Vero cells. Vero cells transfected with VC, WT RTA and RTA mutants were collected at 22 h after transfection. Depurination was measured by qRT-PCR. Fold change is shown relative to the VC. The experiment was repeated four times. Data were analyzed by Fisher’s LSD test for statistical differences. Treatments in group A are statistically different from group B (*P*<0.01). (**C**) mRNA levels of WT RTA and RTA mutants in Vero cells quantified by qRT-PCR at 22 h after transfection. (**D**) Western blot analysis WT RTA and RTA mutants expressed in Vero cells. Total cell lysates were collected at 22 h after transfection. Total protein stain (Supplementary Figure S4B) was used as loading control for the Western blot.

To examine the toxicity and activity of the hydrophobic mutants in Vero cells, the cDNAs encoding the mutant toxins were co-transfected into Vero cells with the EGFP expression plasmid and EGFP fluorescence was measured as an indicator of protein synthesis [39]. The single mutants L232A and R235A showed similar inhibition of protein synthesis as WT (Figure 5A). The double, L232A/R235A and the triple, Y183A/L232A/F240A mutants inhibited protein synthesis significantly less (*P*<0.001) than WT. The quadruple mutant, Y183A/L232A/R235A/F240A did not inhibit protein synthesis and showed same level of protein synthesis as VC (Figure 5A). These results indicated that by combining mutations in critical hydrophobic residues with the R235A mutation, we can eliminate the translation inhibitory activity of RTA in mammalian cells. The depurination activity correlated well with the protein synthesis inhibition and indicated that L232A and R235A depurinated ribosomes at a similar level as WT, but L232A/R235A, Y183A/L232A/F240A and Y183A/L232A/R235A/F240A depurinated significantly less (*P*<0.001) (Figure 5B). The quadruple mutant showed the lowest depurination. The mRNA and protein expression were confirmed by qRT-PCR (Figure 5C) and Western blot analysis, respectively (Figure 5D and Supplementary Figure S4A). Although we did not observe a significant difference in the mRNA levels (Figure 5C), the protein levels showed an inverse correlation with the depurination activity (Figure 5D) based on quantification of the total protein (Supplementary Figure S4B). As observed in yeast, multiple mutations that contained L232A reduced the activity of RTA in Vero cells. The quadruple mutant, which contained R235A, had lower activity than the triple mutant in Vero cells, indicating that it is possible to eliminate the activity of RTA by combining Arg^235^ mutation with mutations in critical hydrophobic residues at the ribosome binding site.

## Discussion

The structural analysis of the RTA–P6 complex identified Tyr^183^, Leu^207^, Phe^240^, Ile^247^, Pro^250^ and Ile^251^ as important residues in the hydrophobic pocket [33,34]. Leu^232^ was not identified as important in either structure although it is part of the hydrophobic pocket [33,34]. We show here that an alanine substitution at Leu^232^ reduced the toxicity and depurination activity of RTA more than alanine substitutions at the other hydrophobic residues in yeast. The sequence alignment of RTA with TCS indicated that Leu^232^ aligns with Leu^215^ in TCS (Supplementary Figure S5A). In the TCS–P11 complex, the C terminally conserved LF motif [15] in P11 was inserted into a hydrophobic pocket lined by Phe^166^, Leu^188^ and Leu^215^ [47] (Supplementary Figure S5B). When RTA–P6 complex was aligned with the TCS–P11 complex, Phe^166^, Leu^188^ and Leu^215^ of TCS were superimposed to Tyr^183^, Leu^207^ and Leu^232^ on RTA, respectively (Supplementary Figure S5A). These data indicate that Leu^232^ is structurally conserved with the stalk binding residues of TCS, suggesting that it plays an important role in stalk binding in RTA.

The single Y183A and F240A mutations caused a significant reduction in the depurination activity in yeast. Although they did not cause a significant reduction in toxicity by themselves (Figure 1C), they reduced toxicity significantly when combined (Figure 2A). L207A also reduced the depurination activity, but not to the same extent as Y183A and F240A. In contrast, the depurination activity was not significantly reduced in yeast expressing V242A, I247A and I251A (Figure 1D). These results demonstrated the functional importance of Leu^232^, Tyr^183^ and Phe^240^ for the full toxicity of RTA in yeast and indicated that Leu^232^, Tyr^183^ and Phe^240^ cumulatively contribute to toxicity with Leu^232^ as the most critical residue in the hydrophobic pocket.

Although point mutations at Arg^235^ and Leu^232^ caused a significant reduction in depurination activity in yeast, neither mutation by itself reduced the depurination activity of RTA in Vero cells. RTA depurinates mammalian ribosomes at a higher rate than yeast ribosomes [48]. Although RTA has similar affinity (*K*_D_) toward yeast [13] and rat liver [49] ribosomes, the catalytic rate (*k*_cat_) of RTA on rabbit ribosomes is five-fold faster (102.5 min^−1^) than the catalytic rate on yeast ribosomes (19.2 min^−1^) [13]. The 50% inhibitory concentration (IC_50_) of peptides corresponding to C-terminal residues of P proteins were six- to ten-times higher for rat ribosomes than for yeast ribosomes [35]. Hence, we deduce that the reduction in activity due to the single L232A or R235A mutations was too small to be observed in Vero cells with any degree of significance. When L232A was combined with R235A the toxicity and depurination activity were reduced in Vero cells (Figure 5A,B). These data indicate that the inhibitory effect of L232A and R235A is additive. When R235A mutation was combined with mutations in residues critical for the hydrophobic interactions (Y183A/L232A/R235A/F240A), a further reduction in protein synthesis inhibition and toxicity was observed. The quadruple mutant, which combined mutations in the critical hydrophobic residues with the R235A mutation (Y183A/L232A/R235A/F240A), eliminated the activity of RTA in Vero cells. We conclude that both hydrophobic interactions and hydrogen bonding with Arg^235^ are critical for the activity and toxicity of RTA. While removing only one type of interaction did not fully abolish toxicity, removing both interactions eliminated the toxicity of RTA in yeast and in mammalian cells. The results obtained with the multiple mutants were consistent between yeast and mammalian cells. The quadruple, Y183A/L232A/R235A/F240A mutation led to a greater reduction in protein synthesis inhibition than the triple Y183A/L232A/F240A mutation in Vero cells, demonstrating that it is possible to distinguish between the RTA mutants with low activity in Vero cells due to the higher catalytic rate of RTA on mammalian ribosomes.

When Y183A and F240A were expressed in yeast (Figure 1E) or in Vero cells (Figure 5D) we did not observe any evidence of degradation, suggesting that their stability was affected in *E. coli*, but not in eukaryotic cells. Protein digestion analysis showed that Y183A and F240A were more sensitive to trypsin digestion compared with WT RTA (Figure 3A). There are two lysine residues, Lys^4^ and Lys^239^, and 21 arginine residues on RTA. Y183A or F240A mutation likely changed the exposure of some of the lysine and arginine residues and led to easier access to trypsin. Y183A and F240A showed multiple bands after the His column purification (Supplementary Figure S3A). Despite the folding defect we were able to purify these mutants to over 90% homogeneity with low yields. We could not detect expression of L232A in *E. coli* (Supplementary Figure S3B) and could not obtain pure protein. Since L232A protein was intact in yeast (Figure 1E) and in Vero cells (Figure 5D) and was toxic in both systems, its folding was affected in *E. coli* but not in eukaryotic cells.

We did not observe detectable binding when we examined the interaction of Y183A and F240A with yeast ribosomes using SPR with a Biacore T200. However, both mutants were able to depurinate ribosomes at a reduced level *in vitro* and in cells, suggesting that alanine substitutions at either hydrophobic residue did not eliminate the interactions with the ribosome. Since RTA reaches the ribosome through electrostatic interactions with the arginine residues especially Arg^235^, the mutant proteins with their intact arginine residues were able to interact with ribosomes even though these interactions were not detected by SPR.

The Y183A and F240A mutants showed reduced depurination activity toward the naked RNA compared with R235A, which depurinated naked RNA as efficiently as WT [15]. These results indicated that alanine substitutions at Tyr^183^ and Phe^240^ reduced catalytic activity of RTA by affecting protein folding. Despite the lower activity toward the naked RNA, Y183A and F240A showed significantly higher depurination activity toward purified ribosomes than R235A. Y183A and F240A were only nine- and three-fold less active on yeast ribosomes than WT RTA, respectively compared with R235A which was 80-fold less active. These mutants were able to depurinate ribosomes *in vivo* and cause toxicity in yeast and in Vero cells. The higher activity of hydrophobic mutants on ribosomes compared with naked RNA suggests that the hydrophobic mutants could partially regain activity in the presence of ribosomes. Ribosomes are thought to be involved in the process of refolding RTA [50]. Partially unfolded RTA could regain full catalytic activity *in vitro* in the presence of salt-washed ribosomes [50]. Ribosome mediated refolding may help hydrophobic mutants to depurinate ribosomes more efficiently in cells. Hydrophobic mutants, which had reduced activity on naked RNA *in vitro*, may be more active and toxic when expressed in eukaryotic cells *in vivo* due to ribosome-mediated refolding. Therefore, the depurination results obtained *in vitro* with ribosomes are consistent with the results obtained in whole cells. The *in vitro* ribosome depurination results may also be more consistent with the toxicity and depurination activity in cells than the *in vitro* results on naked RNA, possibly because *in vitro* ribosome depurination is carried out at pH 7.4, which is similar to the physiological conditions, while *in vitro* RNA depuration occurs at pH 5.

**Figure 6:**
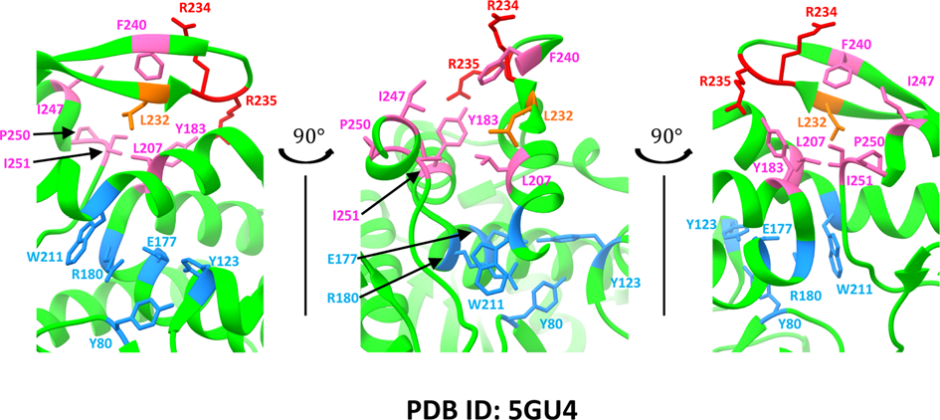
Hydrophobic residues at the stalk binding site are in proximity of the active site residues Structure of RTA (PDB ID: 5GU4) visualized using UCSF Chimera [37]. RTA is colored green. Arginine residues, Arg^234^ and Arg^235^ at the stalk binding site are colored red. Hydrophobic residues, Tyr^183^, Leu^207^, Phe^240^, Ile^247^, Pro^250^ and Ile^251^ are colored hot pink. Leu^232^ is colored orange. Active site residues, Tyr^80^, Tyr^123^, Glu^177^, Arg^180^ and Trp^211^ are colored light blue.

Although R235A retained an intact hydrophobic pocket, it had lower activity on ribosomes than Y183A and F240A, which disrupted the hydrophobic pocket. We previously showed that Arg^235^ serves as an anchor residue at the binding interface because mutations in Arg^235^ reduce the association and the dissociation rate of the interaction with the P stalk [15]. The results obtained here provide further support for this model and suggest that hydrogen bonding with the P stalk mediated through R235A [32–34,51] is more critical than the hydrophobic interactions mediated by Y183A or F240A, possibly because hydrogen bonding brings RTA and the ribosome together, while hydrophobic interactions are important for the stability of the complex [52]. Analysis of the mutations in Tyr^183^ and Phe^240^ described here provides the first evidence that the interaction of P stalk C-termini with the hydrophobic pocket on RTA promotes a conformational rearrangement to correctly position the active site residues for catalytic attack on the SRL. Based on these results, our model can be further described as a ‘latch and lock’ mechanism where Arg^235^ acts as a ‘latch’ for initial protein recognition to promote insertion of the hydrophobic residues at the P-protein C-termini into the hydrophobic pocket. Once Arg^235^ is docked, interactions with Leu^232^, Tyr^183^ and Phe^240^ further contribute to formation of the complex by ‘locking’ the intermediate into the high affinity complex. Tyr^183^ is on an adjacent helix as the active site residues, Arg^180^ and Glu^177^, while Leu^207^ is on the same helix as the active site residue, Trp^211^ (Figure 6). The close spatial proximity of residues in the hydrophobic pocket to the active site residues suggest that insertion of the P-protein C-termini into the hydrophobic pocket on RTA may promote a conformational rearrangement to correctly position the active site residues, Glu^177^ and Arg^180^, in place for catalytic attack on the SRL. This model explains why interaction with the ribosome helps restore the depurination activity of Y183A and F240A mutants on the ribosome, but not the rRNA. Our results indicate that electrostatic interactions precede formation of the hydrophobic interactions. Both interactions need to be eliminated to inhibit the depurination activity and toxicity of RTA in mammalian cells. These results establish the ribosome binding site of RTA as a new target for inhibitor discovery and expand the region of interaction critical for cellular activity to include the hydrophobic pocket. They provide insight into ribosome recognition mechanisms, which may be broadly applicable to other RIPs and translational GTPases which bind to the stalk.

## Supplementary Material

Supplementary Figures S1-S5

## Abbreviations

ANOVA: analysis of variance
CTD: C terminal domain
ER: endoplasmic reticulum
*E. coli*: *Escherichia coli*
mRTA: mature ricin toxin A chain
RIP: ribosome inactivating protein
RTA: ricin toxin A chain
RTB: ricin toxin B chain
SPR: surface plasmon resonance
SRL: α-sarcin/ricin loop
TCS: trichosanthin
VC: vector control/empty vector
WT: wild-type
