# Supplementary material for: Leucine 232 and hydrophobic residues at the ribosomal P stalk binding site are critical for biological activity of ricin
Source: Biosci Rep. 2019 Oct 15;39(10):BSR20192022. doi: 10.1042/BSR20192022 (PMC6822507; doi:10.1042/BSR20192022)
Supplement: Supplementary Figures S1-S5 [file BSR-2019-2022_supp.pdf]

Supplementary Information

Leucine 232 and hydrophobic residues at the ribosomal P stalk binding site are critical for biological activity of ricin

Yijun Zhou, Xiao-Ping Li, Jennifer N. Kahn, John E. Mclaughlin and Nilgun E. Tumer

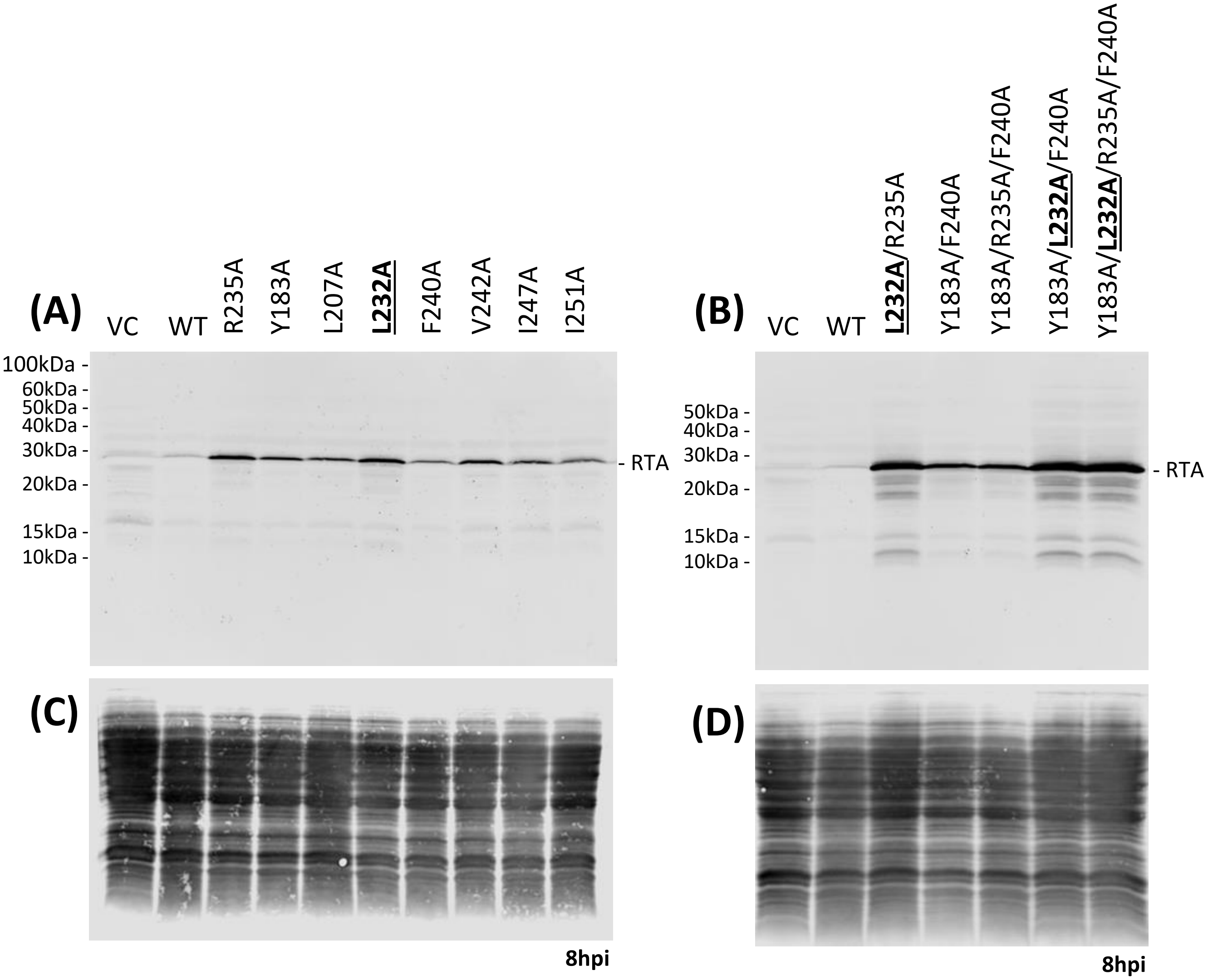

Figure S1. Expression of hydrophobic mutants in yeast.
(A) The full gel of Fig.1E showing western blot analysis of single RTA mutants in yeast at 8 hpi. **(B)** The full gel of Fig. 2C showing western blot analysis of double, triple and quadruple RTA mutants in yeast at 8 hpi. **(C)** Total protein stain of A. **(D)** Total protein stain of B.

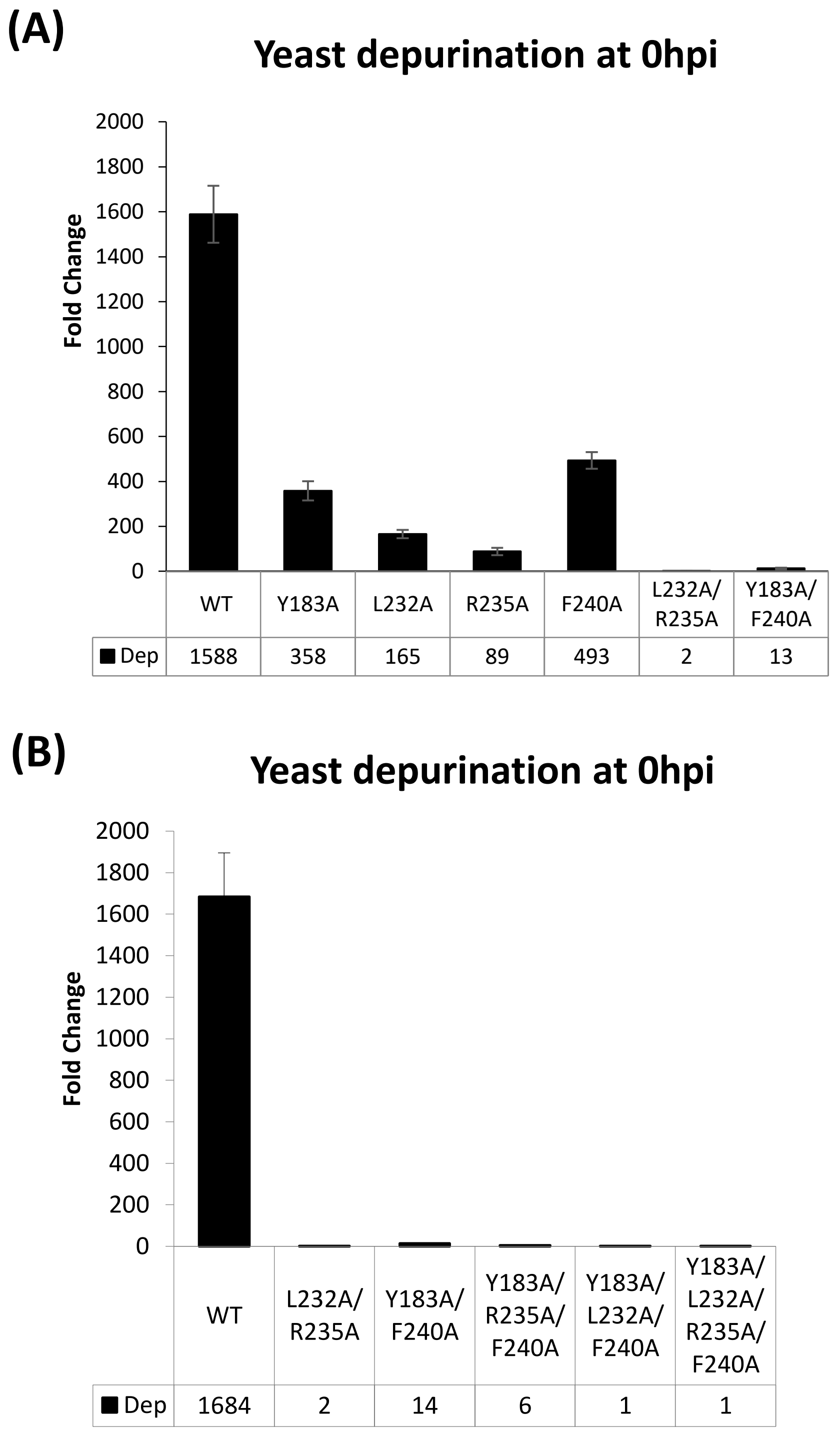

Figure S2. **Depurination of RTA variants in yeast.**
Yeast cells transformed with VC, WT RTA or RTA mutants were cultured overnight in GLU-LEU medium for 20 hours (0 hpi). Depurination was measured by qRT-PCR. **(A)** Depurination of WT RTA, single and double RTA mutants at 0 hpi. **(B)** Depurination of WT RTA, double, triple and quadruple RTA mutants at 0 hpi. Fold change in depurination is shown relative to the VC.

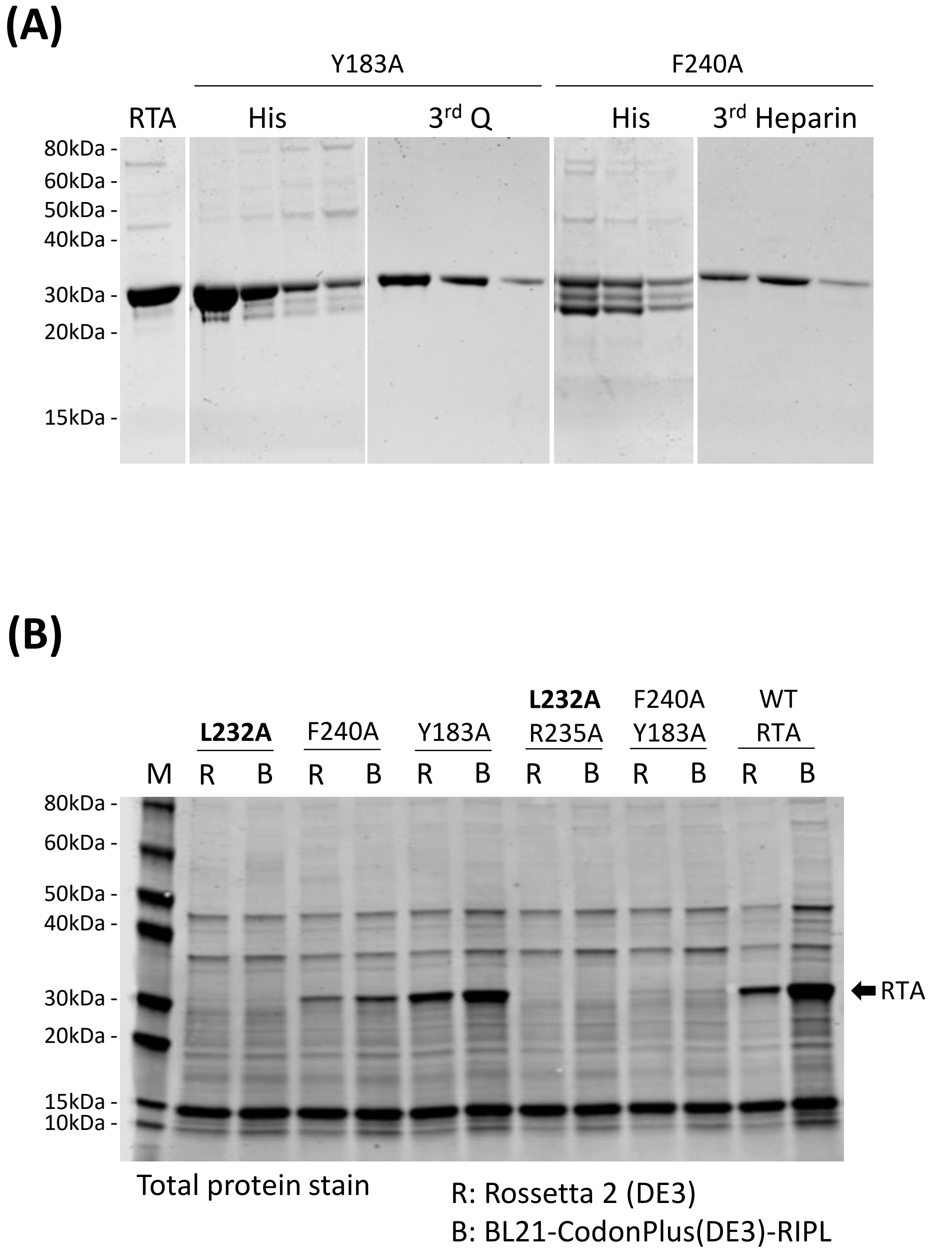

Figure S3. Purification of hydrophobic mutants.
(A) Eluted fractions from His (2nd) and Q (3rd) column purification of Y183A and His (2nd) and Heparin (3rd) column purification of F240A are shown. Purified RTA is loaded as control. **(B)** Hydrophobic mutants were expressed in two different *E.coli* expression strains, Rossetta 2 (DE3) (R) and BL21-CodonPlus(DE3)-RIPL (B), as described in the Materials and Methods. After separation on SDS-PAGE, proteins were transferred to a 0.2 μm nitrocellulose membrane and stained with REVERT total protein stain kit (Li-COR, Lincoln, NE, USA). Molecular weight markers are shown (M).

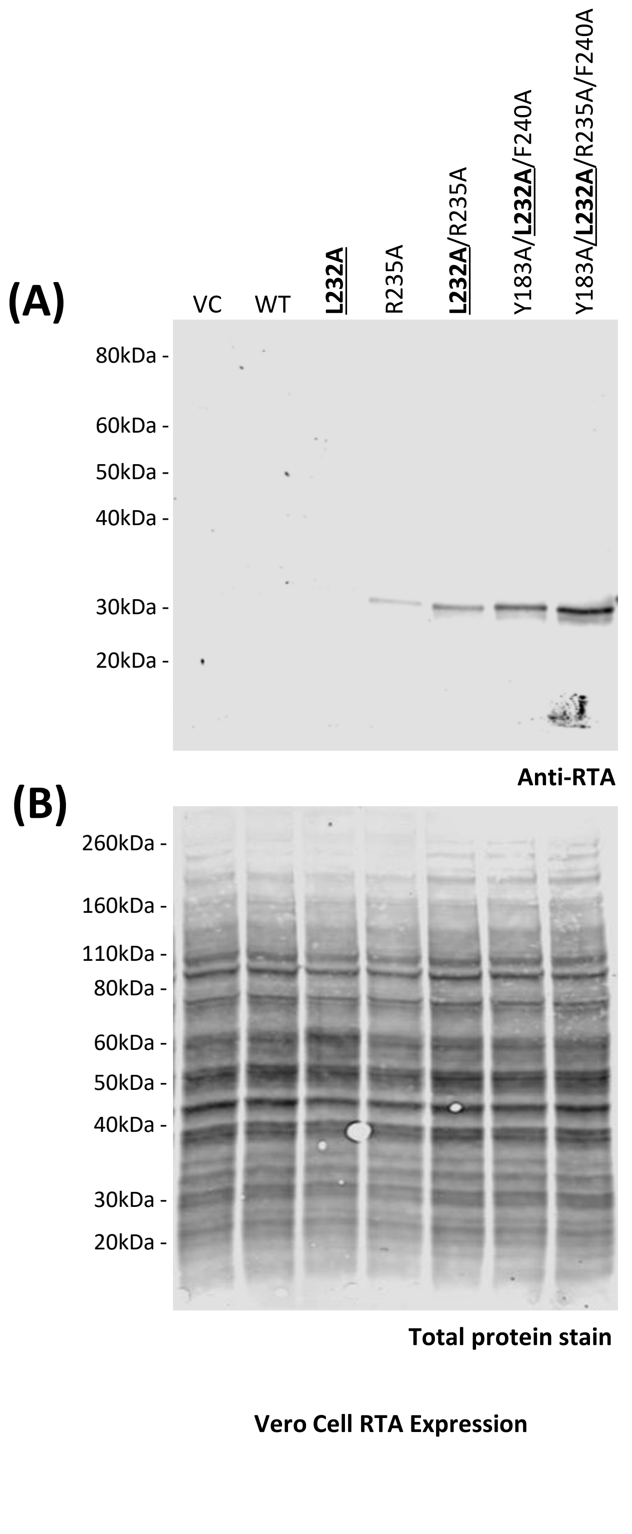

Figure S4. Expression of RTA mutants in Vero cells.
(A) The full gel of Fig. 5D showing western blot analysis of RTA mutants expressed in Vero cells. **(B)** Total protein stain of A.

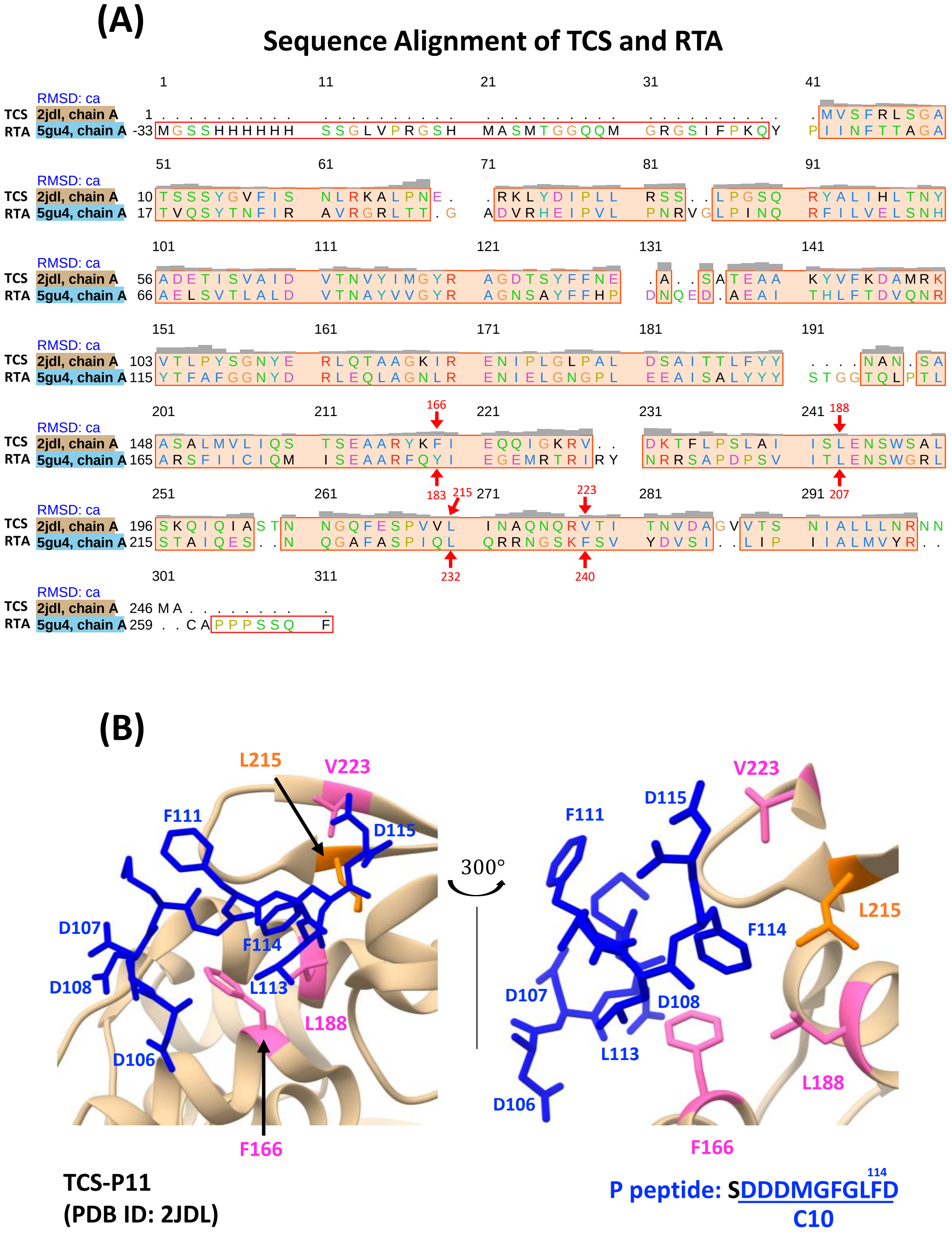

Figure S5. Sequence alignment of TCS with RTA.
(A) TCS (PDB ID: 2JDL) and RTA (PDB ID: 5GU4) were aligned using Chimera. Tyr183, Leu207, Leu232 and Phe240 of RTA and the corresponding residues in TCS, Phe166, Leu188, Leu215 and Val223 respectively, are indicated with arrows and residue numbers. **(B)** Structure of TCS-P11 (PDB ID:2JDL) visualized using Chimera. TCS is colored in tan, Val223, Leu188, and Phe166 are colored in pink. Leu215 is colored in orange. P11 peptide is colored in blue.
